# Olfactory Performance of Children and Adolescents With Type 1 Diabetes: Relationship to Diabetes Duration and Glycemic Control

**DOI:** 10.1155/jdr/8415282

**Published:** 2026-09-14

**Authors:** Grzegorz Sobek, Paweł Jagielski, Artur Mazur

**Affiliations:** ^1^ Faculty of Health Sciences and Psychology, Collegium Medicum, University of Rzeszów, Rzeszów, Poland, ur.edu.pl; ^2^ University Center for Research and Development in Health Sciences, Rzeszów, Poland; ^3^ Department of Nutrition and Drug Research, Institute of Public Health, Faculty of Health Sciences, Jagiellonian University Medical College, Krakow, Poland, cm-uj.krakow.pl; ^4^ Institute of Medical Sciences, Medical College of Rzeszow University, Rzeszow, Poland

**Keywords:** adolescents, glycated hemoglobin, olfactory function, Type 1 diabetes

## Abstract

**Background/Objectives:**

Olfactory dysfunction is increasingly recognized as a complication of diabetes that negatively impacts eating behavior and quality of life. This study investigates olfactory function in adolescents with Type 1 diabetes and evaluates the impact of disease duration and long‐term glycemic control (mean HbA1c) on olfactory performance.

**Methods:**

Olfactory function was assessed using the validated “Sniffin′ Sticks” test, which evaluates odor threshold, discrimination, and identification to generate a composite TDI score for diagnosing hyposmia (TDI < 30.75).

**Results:**

Hyposmia was identified in 30% of adolescents with Type 1 diabetes, whereas none exhibited anosmia. Overall olfactory performance (TDI scores) did not differ significantly by diabetes duration, age, sex, BMI, lipid profiles, or insulin therapy method. However, a strong negative correlation was observed between long‐term mean HbA1c and total TDI scores (*r* = −0.65, *p* < 0.001). Logistic regression confirmed that elevated mean HbA1c (> 7.0%) was a significant independent predictor of olfactory impairment, increasing the risk of hyposmia 3.7‐fold (OR = 3.726, *p* = 0.018).

**Conclusions:**

Poor long‐term glycemic control significantly impairs olfactory function in adolescents with Type 1 diabetes. This is demonstrated by the marked reduction in olfactory scores among adolescents with high mean HbA1c. Consequently, future research may validate olfactory testing as an effective, early biomarker for diabetic complications.

## 1. Introduction

Sensory and metabolic processes mainly determine food selection and consumption. Smell plays an initiating role in both the food interest and consumption phases of eating behavior [1]. Olfactory dysfunction can therefore lead to changes in various aspects of eating behavior, such as food choice, appetite, and food intake [2, 3]. Patients with a diminished sense of smell are less interested in new foods and derive less pleasure from them [4]. Unfortunately, the consequence of losing your sense of smell is not only a reduced pleasure from eating. There is a correlation between olfactory dysfunction in patients and poor diet quality and variability in eating habits [5]. Olfactory dysfunction is common [6] and is also associated with diabetes and especially with diabetic complications [7–9]. Diabetic neuropathy (DN) is a common condition in people with diabetes. It is believed that dysfunction of the sense of smell may be one of the clinical symptoms of central neuropathy [10]. Furthermore, some researchers have also reported that people with diabetic peripheral neuropathy and neuropathic pain have decreased olfactory perception [11]. Other microvascular complications, such as diabetic retinopathy and nephropathy, have also been associated with olfactory dysfunction [10]. The available literature does not allow for a clear association between olfactory function and diabetes and its associated pathologies. However, there are certain trends in the adult population in which the duration of the disease may have influenced this association [8]. In the case of young patients with Type 1 diabetes, studies of olfactory functions, especially in larger groups, are rare. In our previous studies, we observed that long‐term diabetes (> 10 years) in adolescents is associated with poorer taste perception scores, and this may also apply to olfactory function, which we initially assessed using a screening test [12]. Therefore, in this study, we comprehensively assessed the olfactory function of adolescents with short‐ and long‐term diabetes. In our participants, we also assessed the possible impact of long‐term metabolic control markers (mean hemoglobin A1C [HbA1c] value since the disease onset) on olfactory parameters in young patients based on glycated hemoglobin results throughout the disease course.

## 2. Methods

### 2.1. Population

The study comprised a cohort of 113 Caucasian children and adolescents diagnosed with Type 1 diabetes. Participants ranged in age from 10 to 18 years, with a mean age of 13.3 ± 2.0 years. For precise categorization, specific age cut‐offs were recorded down to the exact month. To be eligible for inclusion, patients were required to have a confirmed diagnosis of Type 1 diabetes for a minimum of 5 years. Additionally, documented informed consent from parents or legal guardians was a mandatory prerequisite. Exclusion criteria were strictly applied to maintain the integrity of the sensory evaluations. Individuals born prematurely or with comorbidities such as celiac disease, thyroid disorders, or autoimmune gastritis were excluded from the study. Furthermore, participants were disqualified if they were taking medications known to impair the sense of smell, presented with acute upper respiratory symptoms (such as a runny nose), or suffered from allergies or other medical conditions that could negatively impact olfactory function. Eligible patients were recruited consecutively during their regularly scheduled follow‐up appointments at the clinic of the 2nd Department of Pediatrics, Endocrinology, and Pediatric Diabetology at Clinical Provincial Hospital No. 2 (St. Jadwiga the Queen) in Rzeszów.

### 2.2. Olfactory Tests

Olfactory function was assessed using the validated Extended “Sniffin′ Sticks” test (Burghardt Messtechnik GmbH, Germany). This method evaluates three distinct components of olfaction: odor threshold (T), discrimination (D), and identification (I). During the assessment, stimuli were presented approximately 5 cm from the nose, with an interstimulus interval of at least 20 s to prevent olfactory adaptation.

#### 2.2.1. Odor Threshold

The threshold for n‐butanol was determined using a three‐alternative forced‐choice (3‐AFC) single‐staircase procedure. Patients were presented with triplets of pens at 5‐s intervals; one pen contained the odorant, whereas the other two contained only a solvent. The odorant concentration was increased or decreased based on the patient′s ability to correctly identify the target pen. The test concluded after seven reversals (turning points), and the final threshold score (range 1–16) was calculated as the mean of the last four turning points.

#### 2.2.2. Odor Discrimination

This subtest utilized 16 triplets of pens. In each triplet, two pens contained identical odorants, and one contained a different odorant. Patients were required to identify the odd pen in a forced‐choice format. The score represents the total number of correct responses (range 1–16).

#### 2.2.3. Odor Identification

Patients were presented with 16 common odorants at intervals of approximately 30 s. Prior to each presentation, patients were provided a card with four descriptive terms and asked to select the term that best matched the odor in a multiple‐choice, forced‐choice format. The score reflects the number of correct identifications (range 1–16).

#### 2.2.4. TDI Score

The results of the three subtests were summed to yield a composite TDI score (range 1–48). A TDI score ≥ 30.75 indicates normal olfactory function (normosmia), whereas a score < 30.75 denotes hyposmia [13].

### 2.3. Biochemical Analysis

After a fasting duration of 10 h overnight, blood samples were procured at the laboratory facility of the hospital. Established laboratory methodologies (Siemens Atellica, Germany) were subsequently utilized to evaluate lipid profiles, which included total cholesterol (TC), high‐density lipoprotein (HDL) cholesterol, low‐density lipoprotein (LDL) cholesterol, and triglycerides (TG). The measurement of glycated hemoglobin (HbA1c) was performed using an automated glycohemoglobin analyzer (G8 HPLC, Tosoh Bioscience, Tokyo, Japan).

### 2.4. Analysis of Historical Glycated Hemoglobin Measurements

To evaluate long‐term metabolic control, all historical HbA1c data from the time of diabetes diagnosis were extracted from medical records. Patients had at least one documented HbA1c measurement annually. If 2 or 3 tests were performed within 1 year, the annual average was determined. A final cumulative mean HbA1c was then calculated for each patient, representing their average glycemic control over the entire course of the disease.

### 2.5. Anthropometric Assessment

Participants′ height was measured three times using a portable Seca 213 stadiometer (accurate to 5 mm). Measurements were taken in a standing position, with participants wearing light clothing and no footwear, and the average of the three values was used for analysis. Body weight was assessed to the nearest 0.1 kg using a Tanita BC‐420 body composition analyzer (Tokyo, Japan). Body mass index (BMI) was calculated as weight in kilograms divided by height in meters squared (kg/m^2^). Individual BMI percentiles were then determined using age‐, sex‐, and height‐specific charts developed through the Polish OLAF project (“Developing standards of blood pressure in children and adolescents in Poland”) [14]. Based on these percentiles, participants′ weight status was classified as underweight (< 5th percentile), normal weight (5th to < 85th percentile), overweight (85th to < 95th percentile), or obese (≥ 95th percentile).

### 2.6. Statistical Analysis

Descriptive statistics were computed, encompassing the mean, standard deviation, median, and the first and third quartiles (Q1–Q3). The adherence to the normal distribution of quantitative variables was checked using the Shapiro–Wilk test. To examine the differences among the three groups categorized by T1D duration with respect to the analyzed quantitative variables, the ANOVA test was used. Disparities in qualitative variables across groups were checked using a chi‐square test. Spearman′s rank correlations were used to investigate the interrelations among quantitative variables. Multivariable logistic regression was used to estimate odds ratios (ORs) with 95% confidence intervals (CIs) for the association between HbA1c categories (e.g., 7/≥ 7) and the risk of olfactory impairment. The adjusted model incorporated several covariates that were predetermined: sex, familial history of diabetes (yes/no), and age brackets (10–12/13–17). Statistical analyses were executed using PS IMAGO PRO 10 (IBM SPSS Statistics 29, Armonk, New York, United States). The threshold for statistical significance was established at *p* < 0.05.

## 3. Results

A total of 113 T1D young patients were enrolled. The T1D group comprised 57 boys and 56 girls, with a median age, Me = 13.0 (12.0–15.0). The biochemical and demographic characteristics of the enrolled individuals are reported in Table 1.

**Table 1:** Characteristics of the population.

	T1D group
Age ^∗^	13 (12; 15)
Gender (male/female)	57/56
BMI (percentile grids)	
< 5th percentile (underweight) (%)	1 (0.9)
5th–85th percentile (normal weight) (%)	85 (75.2)
> 85th percentile (overweight or obesity) (%)	27 (23.9)
T–Chol (mg/dL) ^∗^	172 (149; 193)
LDL–Chol (mg/dL) ^∗^	97 (82; 115)
HDL–Chol (mg/dL) ^∗^	60 (53; 70)
TG (mg/dL) ^∗^	73 (58; 104)
HBAIc (%) ^∗^	7.56 (6.84; 8.81)
Duration of diabetes ^∗^	8.0 (6.0; 10.0)
Insulin pomp (*n*, %)	73 (74.5)
Multiple daily injections (*n*, %)	25 (25.5)

∗Median with interquartile range.

### 3.1. Olfactory Tests Results

For the entire study cohort, the median threshold, discrimination, and identification (TDI) score was 34.0 (interquartile range [IQR]: 28.7–37.5). Broken down by subtest, the median scores were 7.5 (IQR: 5.0–10.0) for the threshold test, 12.0 (IQR: 10.0–14.0) for discrimination, and 14.0 (IQR: 13.0–15.0) for identification. Overall TDI scores did not differ significantly by sex (boys: 34.5 [30.0–38.0] vs. girls: 33.4 [28.5–37.4]; *p* = 0.6157). However, boys achieved significantly higher scores on the discrimination subtest compared with girls (13.0 [11.0–14.0] vs. 12.0 [10.0–13.0]; *p* = 0.0306). No sex‐based differences were observed for the threshold test (boys: 7.3 [5.5–10.0] vs. girls: 7.5 [4.8–10.0]; *p* = 0.7648) or the identification test (boys: 14.0 [13.0–15.0] vs. girls: 14.0 [12.0–15.0]; *p* = 0.0852). Based on normative TDI thresholds, 34 adolescents with diabetes (30%) exhibited hyposmia (TDI < 30.75), whereas the remaining 70% fell within the normal olfactory range (normosmia). Notably, no patients presented with anosmia (TDI < 16), and 6 participants (5.3%) achieved exceptionally high scores (TDI > 41.5), classifying them as “supersmellers.”

### 3.2. Diabetes Duration

We found no significant differences between the duration of diabetes and the results of olfactory tests. Total TDI scores were comparable across patients with a diabetes duration of 5–7 years (33.7 [28.7–37.0]), 8–10 years (33.5 [28.5–37.5]), and 10–14 years (35.2 [31.1–38.6]; *p* = 0.8824). Similarly, no duration‐dependent differences were found in the individual subtests. Threshold scores were 7.2 [6.0–9.5] for the 5–7 year group, 7.5 [4.5–10.2] for the 8–10 year group, and 9.2 [4.4–10.9] for the 10–14 year group (*p* = 0.6157). Discrimination scores were 12.0 [10.0–14.0], 13.0 [11.0–14.0], and 13.0 [10.5–13.5], respectively (*p* = 0.8085). Identification scores were 14.0 [13.0–15.0], 14.0 [13.0–15.0], and 14.0 [12.5–15.0], respectively (*p* = 0.7632). Furthermore, diabetes duration was not significantly correlated with mean HbA1c levels. The median HbA1c was 8.0% [6.8–8.8] in the 5–7 year group, 7.4% [6.9–8.7] in the 8–10 year group, and 7.3% [6.8–8.9] in the 10–14 year group (*p* = 0.7813; Table 2).

**Table 2:** T1D duration and TDI scores with diabetes for at least 5 years.

T1D duration		5–7 years	8–10 years	11–15 years	*p* value
Mean HbA1c of T1D duration		7.95 ± 1.32	7.87 ± 1.17	7.75 ± 1.16	*0.7813* ^∗^
		6.1–11.2	6.2–10.9	6.5–10.3	
Threshold test	M ± SD	7.43 ± 2.79	7.58 ± 3.61	8.20 ± 3.70	*0.6157* ^∗^
	Min–Max	2.0–13.5	1.5–14.7	2.0–15.2	
Discrimination test	M ± SD	11.85 ± 2.10	12.15 ± 2.12	12.00 ± 2.29	*0.8085* ^∗^
	Min–Max	7.0–16.0	7.0‐15.0	6.0‐15.0	
Identification test	M ± SD	13.76 ± 1.29	13.74 ± 1.65	13.50 ± 1.90	*0.7632* ^∗^
	Min–Max	12.0–16.0	10.0–16.0	9.0–16.0	
TDI test	M ± SD	33.04 ± 5.16	33.47 ± 5.79	33.70 ± 6.77	*0.8824* ^∗^
	Min–Max	22.0–42.5	21.5–44.2	18.5–44.2	

*Note:* The lack of significance [Table 2] is important from the perspective of the topic of the paper: Olfactory performance of children and adolescents with type 1 diabetes: Relationship to diabetes duration and glycemic control. The results of smell tests are not related to the duration of Type 1 diabetes.

∗ANOVA.

### 3.3. Glycated Hemoglobin

**Figure 1:**
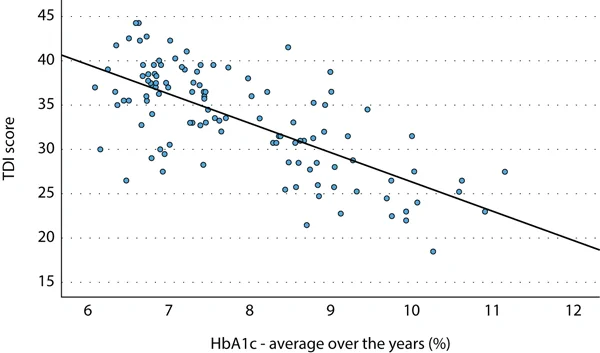
TDI scores according to average HbA1c score since diabetes onset.

**Figure 2:**
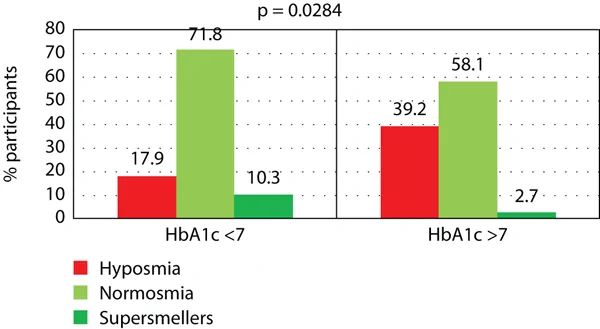
Normosmia and hyposmia depending on the average HbA1c results in adolescents with T1D.

Sperman′s rho assessment demonstrated a strong negative correlation was observed between the long‐term mean HbA1c and total TDI scores (*R* = −0.65, *p* < 0.001) (Figure 1). Logistic regression analysis confirmed that elevated HbA1c levels significantly increased the risk of olfactory impairment (OR = 3.726, 95% CI: 1.258–11.030, *p* = 0.018; Table 3). Specifically, a mean HbA1c > 7.0*%* was associated with a 3.7‐fold increased risk of hyposmia. Hyposmia was present in 39.2% of adolescents with higher mean HbA1c levels, compared with only 17.9% of those with lower mean HbA1c levels (Figure 2).

**Table 3:** Logistic regression analysis for olfactory function (TDI score).

Variable	*β*	SE	Wald	*p* value	OR	95% Cl
Gender	0.465	0.463	1.009	0.315	1.593	0.642–3.948
HbA1c	1.315	0.554	5.645	0.018	3.726	1.258–11.030
DitF	0.405	0.508	0.637	0.425	1.500	0.554–4.056
Age	0.737	0.496	2.209	0.137	2.089	0.790–5.518

*Note:* Gender: women/men; HbA1c (mean HbA1c of T1D duration): < 7/≥ 7; DitF (diabetes in the family): yes/no; age (years): 10–12/13–17.

### 3.4. Biochemical and Demographic Variables

A Mann–Whitney *U* test revealed a significant difference in long‐term mean HbA1c between younger (10–12 years) and older (13–17 years) adolescents, with the older cohort exhibiting higher average HbA1c levels (7.6% [6.9–8.9] vs. 7.4% [6.7–8.5]; *p* = 0.0483). Despite this, logistic regression analysis did not identify age as a significant risk factor for olfactory disorders (OR = 2.089, 95% CI: 0.790–5.518, *p* = 0.137). Similarly, sex was not a significant predictor of olfactory impairment (OR = 1.593, 95% CI: 0.642–3.948, *p* = 0.315; Table 3). Further analysis confirmed no significant correlations between olfactory test results and other clinical or demographic variables, including age, sex, BMI, or diabetes duration. Additionally, no significant effects on olfactory function were detected for other metabolic parameters, including TC, LDL cholesterol, HDL cholesterol, and TG (Table 4). Finally, the mode of insulin therapy did not significantly influence olfactory outcomes. Median TDI scores were comparable between patients using insulin pumps (35.3 [30.0–38.5]) and those using insulin pens (34.5 [30.5–36.5]; *p* = 0.0912).

**Table 4:** Correlation between various demographic and biochemical parameters and olfactory function.

	Rs	*p* value
Age	0.08	0.408 ^∗^
BMI (percentile grids)	−0.01	0.886 ^∗^
T–Chol (mg/dL)	0.02	0.859 ^∗^
LDL–Chol (mg/dL)	0.01	0.915 ^∗^
HDL–Chol (mg/dL)	0.05	0.613 ^∗^
TG (mg/dL)	−0.11	0.228 ^∗^
Duration of diabetes	0.06	0.509 ^∗^

∗Spearman′s rank correlation analysis.

## 4. Discussion

Type 1 diabetes is potentially associated with serious microvascular and macrovascular complications [3, 11, 15, 16], although they are usually subclinical in childhood and adolescence. The duration of diabetes, glycemic control, age, and puberty are key factors contributing to the development of such problems. Other risk factors include smoking, hypertension, higher BMI, and dyslipoproteinemia [16]. Importantly, diabetic complications, such as peripheral DN, as well as other microvascular complications, such as diabetic retinopathy and nephropathy, are associated with olfactory dysfunction [3, 8, 11, 16–18]. The likelihood of their occurrence increases with the duration of diabetes and sometimes the severity of hyperglycemia. DN has been estimated to occur in approximately half of all children with Type 1 diabetes with a duration of 5 years or longer, and up to 25% of pediatric patients with newly diagnosed diabetes have abnormal findings on nerve conduction studies (NCS) [19, 20]. Regarding diabetic retinopathy, a leading cause of preventable blindness, studies consistently report a prevalence of around 80% for any stage of the condition in T1DM patients after 5 years of disease progression [21]. The main risk factor for developing late complications of Type 1 diabetes, including neuropathy, is prolonged disturbances in glycemia [22–25]. Research results indicate that the most frequently mentioned variables for the occurrence of various complications in diabetics include the duration of diabetes [26, 27].

Goals aimed at reducing the risk of microvascular and macrovascular complications in children and adolescents with T1DM include HbA1c < 7.0*%*, normal lipid levels, blood pressure < 90th percentile for age, sex, and height, BMI < 95th percentile, no smoking, and physical activity [28, 29].

HbA1c is used as an index of average blood glucose (BG) measurement over a period of months and is a mainstay of BG monitoring. This metric is easy to measure and relatively inexpensive to obtain, and it predicts diabetes‐related microvascular complications [30]. High HbA1c levels contribute to structural deficits in the olfactory system. Among patients with Type 2 diabetes, this manifests as significant variations in olfactory bulb volume, tract length, and sulcus depth [31]. However, HbA1c alone provides an incomplete assessment of glycemic control, as it does not reflect short‐term fluctuations in BG levels known as glycemic variability (GV) [32]. Conversely, elevated HbA1c levels are associated with a greater mean amplitude of 24‐h glycemic fluctuations compared with lower or moderate HbA1c ranges [33]. Lazar et al. [34], demonstrated a moderate but significant inverse correlation between time in range (TIR) and HbA1c, reinforcing that increased time in the optimal glycemic range translates to lower HbA1c levels.

In this paper, we examined how the duration of diabetes and glycemic control affect olfactory function in young individuals with diabetes. Consequently, it is expected that the prevalence of olfactory dysfunction in patients with diabetes correlates with longer disease duration and the severity of hyperglycemia [35]. Our previous research using the U‐Sniff test indicated that a diabetes duration exceeding 10 years was associated with poorer olfactory performance [12]. However, the current study, utilizing a more accurate and standardized tool—the Sniffin′ Sticks test (SST), (Burghart Messtechnik)—failed to confirm these earlier findings. Diabetes duration (5–7, 8–10, or 11–14 years) had no significant impact on the olfactory test results. The results of all tests and their sum in the form of the TDI score were similar. It is important to note that the duration of diabetes per se does not inevitably lead to diabetic complications or associated symptoms like olfactory impairment. A notable aspect of this study was the comparison of olfactory performance with longitudinal HbA1c data spanning the entire course of the disease. The high frequency and regularity of testing in young patients enabled a comprehensive reconstruction of their glycemic control from the onset of diabetes.

Our findings demonstrate an inverse relationship between average HbA1c levels and overall TDI scores; specifically, higher historical HbA1c levels were associated with poorer olfactory function in young patients with Type 1 diabetes. Clinically, the standard target for optimal therapeutic efficacy in diabetes mellitus (DM) is maintaining an HbA1c level below 7.0% [36]. Our results indicate that patients with mean HbA1c levels below 7.0% were more likely to achieve normative TDI scores. In this group, only 18% had total scores below the 30.75 threshold indicative of hyposmia. These findings align with published normative data for the Sniffin′ Sticks test. In a large cohort of healthy individuals aged 11–20 years, the prevalence of hyposmia was reported to be 19.5%, which is remarkably similar to the 18% observed in our well‐controlled diabetic cohort (HbA1c < 7.0*%*) [37]. Conversely, among young patients with mean HbA1c levels above 7.0%, the prevalence of hyposmia was notably higher, exceeding 39%.

According to certain studies, nearly half of adult patients with Type 1 diabetes experience hyposmia or anosmia [38]. Furthermore, the decline in SST performance seen in this population showed no correlation with other evaluated factors, such as age, gender, BMI, family history of the disease, diabetes duration, or the specific insulin regimen used. Subsequent regression analyses of these variables verified that the long‐term average HbA1c level was the only independent predictor of olfactory functions. However, relying solely on HbA1c is suboptimal, as it fails to capture GV—a crucial predictor of micro‐ and macrovascular complications [39]. The absence of GV data in our analysis likely explains why disease duration did not significantly associate with olfactory decline. Ultimately, patients who maintain more stable BG levels may experience a delayed onset or complete avoidance of such complications.

Chronic poor glycemic control is a known catalyst for the rapid onset of DN [40]. Consequently, the early diagnosis of such complications in pediatric and adolescent populations with diabetes is of utmost importance. Although NCS represent the gold standard for identifying subclinical DN, their clinical utility is limited by their invasive nature, technical difficulty, and restriction to assessing only large nerve fibers [41]. To address these limitations, sensory testing has been suggested as an adjunctive diagnostic tool [15, 42]. Although olfactory evaluation cannot substitute for a comprehensive clinical examination [9, 11], it offers a simple and practical means of screening olfactory function, potentially serving as an early indicator of diabetic complications like neuropathy. This is confirmed by our study, which demonstrates a link between high glycated hemoglobin levels and hyposmia, one of the recognized signs of diabetes complications. Evaluating olfactory function has substantial diagnostic importance. Olfactory disorders are widely recognized for their marked effects on quality of life because they impair nonverbal communication and diminish individuals′ general well‐being. They have also been linked to symptoms of depression [43, 44]. Moreover, the interrelationships among the senses are well established; for example, in adolescents, abnormal findings on olfactory testing have been associated with an increased risk of taste disorders [45]. Olfactory dysfunction is often associated with changes in eating behavior and compromised nutritional status [43, 46, 47]. It has further been demonstrated that people with reduced smell are less inclined to show interest in new foods and experience less enjoyment from eating [48]. At the population level, an association has been observed in patients with olfactory disorders between poor diet quality and reduced dietary diversity [5]. In addition, because the sense of smell shapes food intake behaviors, alterations in dietary habits and/or food preferences that accompany olfactory disorders may influence metabolic control, with potential long‐term effects on the natural history of DM [49]. Given these relationships, clinicians are encouraged to monitor olfactory and gustatory function more closely, as identifying deficits may warrant additional diagnostic assessment of cognitive function, nutritional status, and thyroid health, and also provide an opportunity to counsel patients on required lifestyle interventions.

A key strength of the study is its cohort size: It included 113 participants, representing 20% of patients with Type 1 diabetes aged 10–18 in the Podkarpacie region, thereby enhancing the reliability and representativeness of the findings. The demonstrated high prevalence of hyposmia in individuals with elevated glycated hemoglobin levels underscores the potential impact of this condition on dietary choices and points to directions for future research into its behavioral and nutritional changes.

Our results should be interpreted in light of several limitations. First, due to the cross‐sectional design, causal relationships cannot be inferred from the observed associations. Second, the findings may not be generalizable to all children and adolescents with Type 1 diabetes, as the cohort was a convenience sample recruited from a single pediatric center. In addition, some other important factors were not considered, including pubertal assessment. Third, although beyond the scope of our aims, we did not assess GV, a factor that significantly influences diabetic complications and outcomes, nor did we analyze other CGM parameters. Finally, the frequency of annual HbA1c measurements varied among patients, potentially affecting the representativeness of these values for individual participants. Furthermore, the absence of established olfactory test norms for healthy children and adolescents makes it difficult to interpret results obtained from patients with diabetes.

## 5. Conclusion

Our findings demonstrate that long‐term glycemic control, rather than the mere duration of diabetes, is the critical factor requiring vigilant monitoring to prevent complications in adolescents with Type 1 diabetes. This is evidenced by the significantly reduced olfactory function scores observed in young patients with elevated mean HbA1c levels. Moving forward, further investigation into olfactory testing may establish it as a beneficial method for the early identification of diabetic complications.

## Author Contributions

Grzegorz Sobek: conceptualization, methodology, resources, investigation, writing—original draft, data curation, formal analysis, and writing—review and editing. Paweł Jagielski: software, visualization, data curation, formal analysis, and writing—review and editing. Artur Mazur: resources, supervision and project administration. All authors have read and agreed to the published version of the manuscript.

## Funding

No funding was received for this manuscript.

## Disclosure

All authors have read and approved the final version of the manuscript. The corresponding author had full access to all of the data in this study and takes complete responsibility for the integrity of the data and the accuracy of the data analysis.

## Ethics Statement

The study process was in accordance with the Declaration of Helsinki and was approved by the institutional Bioethics Committee at the University of Rzeszow on March 14, 2019 (Resolution No. 9), and by all relevant administrative bodies. Written informed consent was obtained from the participants′ legal guardians or next of kin. We obtained assent from all study participants.

## Conflicts of Interest

The authors declare no conflicts of interest.

## Data Availability

The data that support the findings of this study are available from the corresponding author upon reasonable request.
